# Research progress on gas signal molecular therapy for Parkinson’s disease

**DOI:** 10.1515/biol-2022-0658

**Published:** 2023-08-10

**Authors:** Linlin Wang, Qing Dan, Bingxuan Xu, Yun Chen, Tingting Zheng

**Affiliations:** Department of Hubei University of Medicine, Shenzhen Key Laboratory for Drug Addiction and Medication Safety, Institute of Ultrasonic Medicine, Peking University Shenzhen Hospital, Shenzhen Peking University-Hong Kong University of Science and Technology Medical Center, Shenzhen 518036, P. R. China

**Keywords:** Parkinson’s disease, hydrogen sulfide, nitric oxide, carbon monoxide, hydrogen

## Abstract

The pathogenesis of Parkinson’s disease (PD) remains unclear. Among the pathological manifestations is the progressive degeneration of the nigrostriatal dopaminergic pathway, leading to massive loss of neurons in the substantia nigra pars compacta and dopamine (DA) depletion. Therefore, the current drug treatment is primarily based on DA supplementation and delaying the progression of the disease. However, as patients’ symptoms continue to worsen, the drug effect will gradually decrease or even disappear, thereby further aggravating clinical symptoms. Gas signaling molecules, such as hydrogen sulfide (H_2_S), nitric oxide (NO), carbon monoxide (CO), and hydrogen (H_2_), exhibit pleiotropic biological functions and play crucial roles in physiological and pathological effects. In common neurodegenerative diseases including Alzheimer’s disease and PD, gas signal molecules can prevent or delay disease occurrence via the primary mechanisms of antioxidation, anti-inflammatory response, and antiapoptosis. This article reviews the therapeutic progress of gas signaling molecules in PD models and discusses the possibility of their clinical applications.

## 1 Introduction and research status of Parkinson’s disease (PD)

PD is a common neurodegenerative disease [1], which is primarily manifested as resting tremors, stiffness, slowness, and other motor symptoms, along with non-motor symptoms, such as cognitive decline, emotional depression, and anxiety [2,3]. PD often occurs in elderly individuals and mostly in men. The number of PD cases is estimated to double from 2015 to 2040 because of global aging [4,5]. PD progresses slowly and worsens progressively, from local motor tremors at the start of onset to long-term symptoms rendering the patient bedridden, which seriously affects the patient’s quality of life and life expectancy. There is a substantial increase in the number of patients, undoubtedly increasing the domestic and global economic burden [6]; therefore, it is urgent to prevent and treat PD.

The pathogenesis of PD remains unclear, and the primary pathological manifestation is the progressive degeneration of the nigrostriatum, leading to a considerable loss of neurons in the substantia nigra pars compacta (SNpc) and dopamine (DA) depletion [6,7]. Abnormally folded α-synuclein (α-SYN) is accumulated in the cytoplasm of residual neurons in the substantia nigra, forming eosinophil Lewy bodies [8], which in turn induces dysfunction of DA synthesis.

PD pathogenesis may be related to several factors, including apoptosis, inflammatory reactions, and oxidative stress (OS) [9,10]. Apoptosis of DA-containing neurons in the SNpc of patients with PD has been reported, along with the subsequent induction of dysfunction of DA synthesis [10]. Therefore, the study of apoptosis in PD pathogenesis is of great value. Activated microglia have been observed in substantia nigra of patients with PD, and inflammatory cytokines, including reactive oxygen species (ROS) and tumor necrosis factor (TNF-α) [11,12], have been found to significantly increase, indicating that the inflammatory response is involved in PD progression. Research has revealed that 1-methyl-4-phenyl-1 (MPTP) [13] and 6-hydroxydopamine (6-OHDA) [14] are commonly used to construct PD cell and mouse models. MPTP and 6-OHDA can induce an increase in ROS in dopaminergic neurons, resulting in neuronal death [15]. Simultaneously, it can also promote the release of proinflammatory factors, such as reactive oxygen-free radicals [16], causing neuroinflammation and eventually resulting in the death of DA neurons.

Among various factors, OS is closely related to the pathogenesis of PD [17].

**Figure 1:**
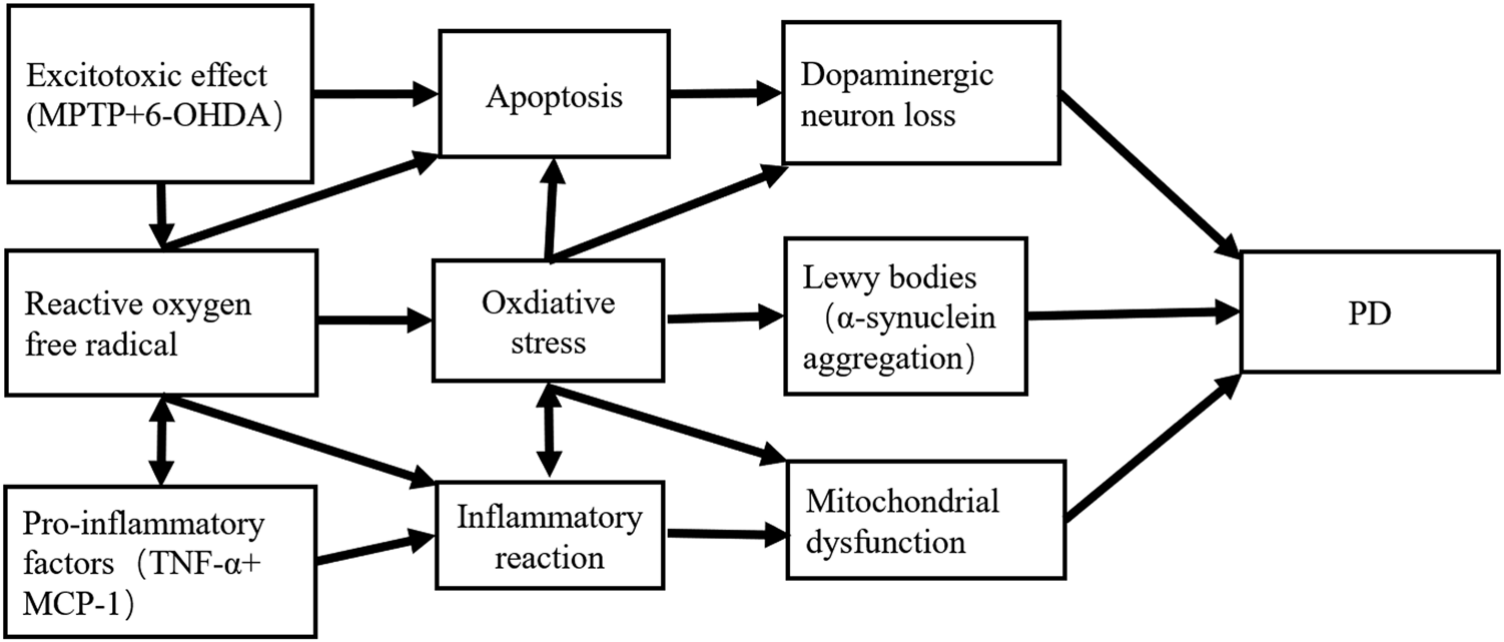
Main pathological mechanism of PD. The relationship between OS, apoptosis, and inflammatory response and the main pathological mechanism of PD [20].

Under various external conditions, OS causes an imbalance between oxidation and antioxidation in the body, abnormal intracellular metabolism, excessive production of ROS substances, oxidative DNA damage, cytotoxicity, destruction of different molecular and cellular structures, and alterations in the functions of organs and systems [18]. Neurons are more sensitive to OS than other cells because of their large oxygen demand and relatively few antioxidants; thus, they are extremely vulnerable to ROS damage [19]. Studies have reported that the levels of oxidized proteins in the substantia nigra of patients with PD are higher than that of normal controls; therefore, DA neurons may be deformed because of endogenous OS. Studies have reported a mutual regulation between ROS and many inflammatory factors. The long-term existence of ROS activates inflammatory cells, leading to the production of various proinflammatory factors, including TNF-α and monocytechemotacticprotein-1 [11]. Activated inflammatory factors can increase ROS production, indicating a positive feedback regulation mechanism between OS and inflammatory response (Figure 1).

The primary purpose of PD treatment is to relieve symptoms and delay disease progression. The current PD treatment primarily includes drug therapy, surgical treatment, food, exercise, and other adjuvant treatments [21]. Among these treatments, drug therapy is the primary treatment method, providing the best relative effect. However, as the disease progresses, the therapeutic effect gradually decreases or even disappears, thereby further aggravating clinical symptoms. The common goal of scientists at home and abroad is to explore better PD treatment methods, along with novel drugs and delivery methods, and thus, more targeted treatments for PD have been developed. Therefore, this article reviews the therapeutic progress of gas signaling molecules in PD models and discusses the possibility of their clinical applications.

## 2 Introduction to gas signaling molecules

Gas signaling molecules are gaseous molecules with short half-lives [22], including hydrogen sulfide (H_2_S), nitric oxide (NO), carbon monoxide (CO), and hydrogen (H_2_). The abovementioned gases were previously known by the characteristics of the gas itself, among which NO and H_2_S have been identified as harmful gases [23]. Recently, studies have revealed that gas signaling molecules exhibit pleiotropic biological functions. They are endogenously produced in organisms and play crucial roles in cellular homeostasis [24]. These gases exhibit antioxidative, anti-inflammatory, and antiapoptotic properties; thus, they play corresponding roles in the treatment and prevention of various diseases [22].

**Figure 2:**
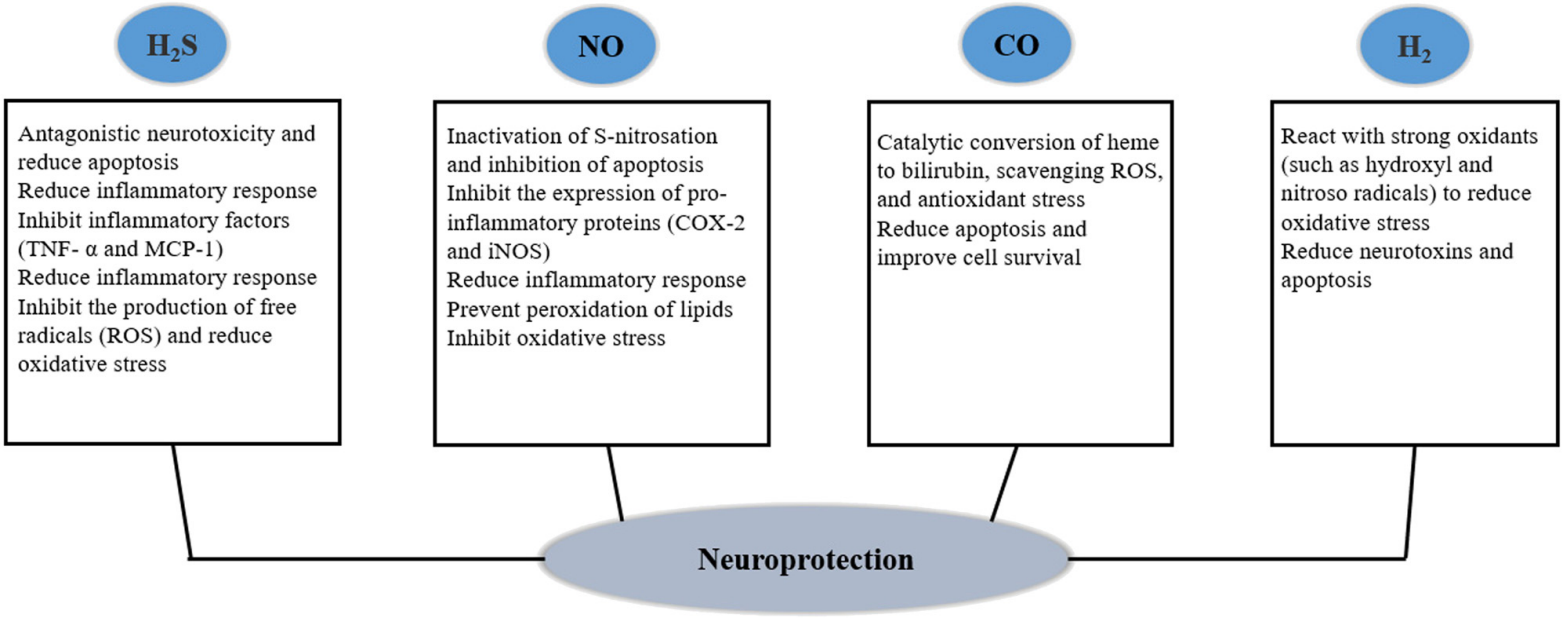
The potential mechanism of gas signal molecules in neuroprotection [29].

Gas signaling molecules can participate in the regulation of various reaction processes in cardiovascular system diseases; reducing blood pressure by dilating blood vessels, regulating monocytes and endothelial cells, and performing other related activities to reduce inflammatory reactions [25]. In the urinary system, these molecules can delay kidney disease development and improve the physiological functions of the body via antifibrotic mechanisms [26]. In various malignant tumors, including breast cancer, colon cancer, ovarian cancer, and other malignant diseases, gas signaling molecules regulate the homeostasis of the cells or body to enhance apoptotic stimulation and inhibit metastasis and angiogenesis processes [27]. They also exhibit physiological effects on the immune, reproductive, and digestive systems [28]. In common neurodegenerative diseases including Alzheimer’s disease and PD, gas signal molecules can prevent or delay disease occurrence via the primary mechanisms of antioxidation, anti-inflammatory response, and antiapoptosis [29]. Compared with PD symptomatic treatment that primarily supplements DA with drugs, gas signaling molecules exert therapeutic effects by blocking the relevant underlying mechanisms, and therefore, these molecules have valuable research significance (Figure 2).

## 3 Application of H_2_S in PD

### 3.1 Synthesis and biological functions of H_2_S

There are four *in vivo* biological enzyme pathways for H_2_S synthesis [22,26], including cystathionine β-synthase (CBS), cystathionine γ-lyase (CSE), 3-mercaptopyruvate sulfur transferase (3MST), and half cysteine aminotransferase. The pathway 3MST is coupled with peroxisome-located d-amino acid oxidase. These catalytic enzymes are distributed in various system tissues; CBS expression has been reported in the nervous system, medium and high CSE expression is primarily observed in the cardiovascular system, and the expression of both these enzymes is noted in the liver and kidney. H_2_S is abundant in the body and has several production pathways. Based on extensive research, in the central nervous system, H_2_S reduces the damage of DA neurons in the substantia nigra via various mechanisms, including antioxidation and anti-inflammation. In PD, Alzheimer’s disease, and other neurodegenerative diseases [30], H_2_S may exert therapeutic effects by delaying disease progression. In a previous study, the intraocular pressure was found to significantly reduce after the injection of sodium hydrosulfide (NaHS, a donor of H_2_S) into the vitreous cavity of glaucoma rat models [31]. The results revealed that the experimental rats exhibited lower retinal degeneration and fewer apoptotic cells than the blank group. Western blotting revealed that exogenously administered H_2_S decreased the expression of P53, Bax, and caspase-3 in rat models, reducing cellular apoptosis and protecting nerve cells [32].

### 3.2 Protective effects of H_2_S and its application in PD

**Figure 3:**
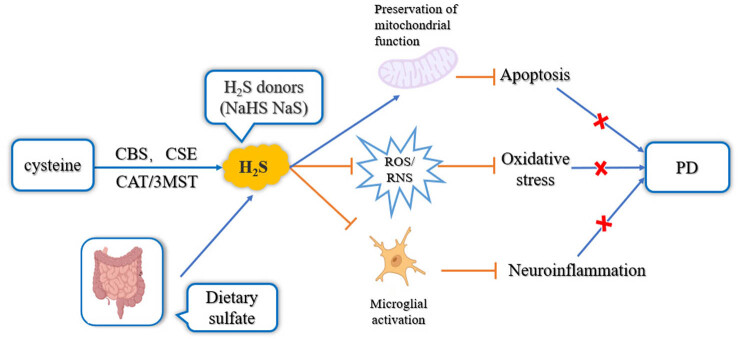
The endogenous and exogenous sources of H_2_S and the main mechanism of its protective role in PD [38,39]. H_2_S prevents the onset and progression of PD by inhibiting the production of ROS, the activation of microglial and promoting the repair of mitochondrial function (activation 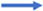; inhibition 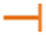).

In 1996, Abe and Kimura [33] reported for the first time that H_2_S is an endogenous neuronal regulator. In a follow-up study, in 2010, Hu et al. [30] established a PD rat model induced by 6-OHDA and rotenone. 6-OHDA was the first discovered DA neurotoxin and has been applied in PD models [14]. The primary pathogenic mechanism underlying the PD model constructed by 6-OHDA is the massive OS induced by the toxin, causing neuronal damage [33]. Rotenone inhibits mitochondrial complex 1, and its primary underlying pathogenic mechanisms include the loss of DA neurons and inflammatory responses in the DA pathway [34]. Studies have revealed that in the abovementioned two PD animal models, the regular administration of a corresponding amount of NaHS can attenuate symptoms caused by the two neurotoxins, reducing the number of apoptotic cells, and delaying PD progression [35]. H_2_S inhibits neuronal apoptosis in the substantia nigra by promoting 6-OHDA-induced leptin signaling in PD rats. In a PD rat model established using rotenone, NaHS attenuated cognitive impairment and promoted the activation of microglia from M1 to M2 in the hippocampus. M1 microglia release inflammatory factors to destroy neurons, while M2 microglia can release anti-inflammatory factors, cytogenic factors, and neurotrophic factors, thus playing an anti-inflammatory and neuroprotective role [36]. The warburg effect, also known as aerobic glycolysis, refers to the process of glycolysis under aerobic conditions. The Warburg effect plays an important regulatory role in brain function, promoting the maturation of neurons during postnatal development. H_2_S was found to inhibit cognitive dysfunction in PD by enhancing the hippocampal Warburg effect and promoting the M2 polarization of microglia. In an MPTP-induced PD mouse model, NaHS has been reported to antagonize MPTP-induced neurotoxicity and also protect nerve cells [37]. These results reveal that H_2_S exhibits neuroprotective properties to treat and prevent neurotoxin-induced neurodegeneration via multiple mechanisms such as antioxidation, anti-inflammation, and metabolic inhibition; thus, H_2_S has potential therapeutic value for PD treatment (Figure 3).

In various experiments reporting the biological function of H_2_S, the commonly used H_2_S donors include NaHS, NaS, and calcium sulfide [40]. H_2_S donor administration and direct inhalation are the two common methods of H_2_S administration [41]. However, this method has certain limitations *in vivo*. The H_2_S donor is instantaneously released in the mouse model with a quick degradation rate. Compared with the slow and continuous release process in the body, the experimental results using the donor will not be accurate enough, and it will induce harmful effects causing damage to the human body. The inhalation of H_2_S is a feasible method along with the donor administration of H_2_S. However, H_2_S has the smell of rotten eggs and is an irritant [42]. It is difficult to tolerate the inhalation of even a small amount of low-concentration H_2_S. Therefore, current research has been focused on identifying a method for H_2_S delivery. Ultrasonic microbubble gas signaling molecules, such as NO and CO, can achieve be directly delivered to improve the therapeutic effects. H_2_S, which is also a gas signaling molecule, can be directly delivered in combination with ultrasonic microbubbles for treatment. Studies revealed that ultrasonic microbubbles carry H_2_S to form H_2_S microbubbles, releasing H_2_S in the myocardium and playing an effective role in reducing myocardial ischemia–reperfusion in rats; moreover, it has no obvious or systemic adverse effects [43,44]. However, microbubbles are currently mainly used in cardiovascular models, and they remain to be tested in PD models. Therefore, more extensive studies are needed to explore their effects.

## 4 Application of NO in PD

### 4.1 Formation and physiological functions of NO

**Figure 4:**
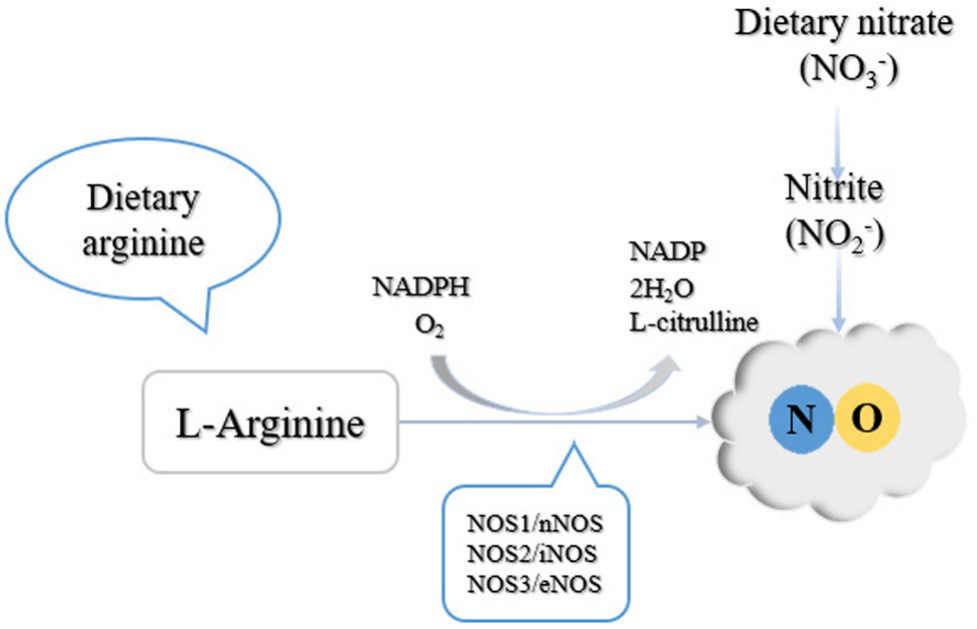
Endogenous and exogenous pathways of nitric oxide production; NADPH indicates nicotinamide adenine dinucleotide phosphate [20]. NADPH indicates nicotinamide adenine dinucleotide phosphate.

NO can be generated through endogenous and exogenous pathways [45]. The endogenous pathway uses NOS-mediated catalysis of the interaction between the substrate arginine and oxygen through a series of processes to obtain NO. NOS includes three subtypes, namely neuronal nitric oxide synthase (nNOS), inducible nitric oxide synthase (iNOS), and endothelial nitric oxide synthase (eNOS). NO catalyzed by different types of NOS has different physiological functions [46]. NO produced by nNOS is responsible for the signal transduction of neurons in the central nervous system and the relaxation of vascular and non-vascular smooth muscles in the peripheral nervous system [47]. NO catalyzed by iNOS is responsible for the immune response through the defense mechanism of macrophages [48]. Furthermore, NO derived from eNOS relaxes the blood vessels, inhibits the adhesion of platelets and leukocytes, and regulates the expression of the vascular endothelial growth factor [49]. Exogenous pathways obtain NO through the reduction of food by nitrite reductase. The main underlying mechanism is as follows: nitrite is first protonated into HNO_2_^−^ in the stomach and then decomposed into nitrogen oxides, including NO [50]. Foods containing l-arginine, such as meat, seafood, and nuts, are the main sources of exogenous NO supplementation. l-Arginine is directly involved in the synthesis of NO [51]. l-Citrulline is the precursor of l-arginine, which is further converted into NO through the catalytic reaction of NOS and mainly occurs in the kidney [51] (Figure 4).

### 4.2 Dual roles of NO in PD

Among the various pathogenic factors of PD identified so far, including age, excessive oxidation reaction, mitochondrial dysfunction, and exogenous neurotoxins, NO plays a crucial role in many pathways that cause PD [52]. iNOS can participate in a defense mechanism with immune cells, oxidative reactions, and inflammatory non-immune nerve cells. Furthermore, iNOS overexpression can increase the production of NO and initiate or increase the process of inflammation and OS. Excess NO can inhibit the associated respiratory complexes. The depletion of the respiratory chain and glutathione (GSJ) triggers mitochondrial dysfunction [53], which results in gradual irreversible damage to nerve cells. This leads to the development of related neurodegenerative diseases.

**Figure 5:**
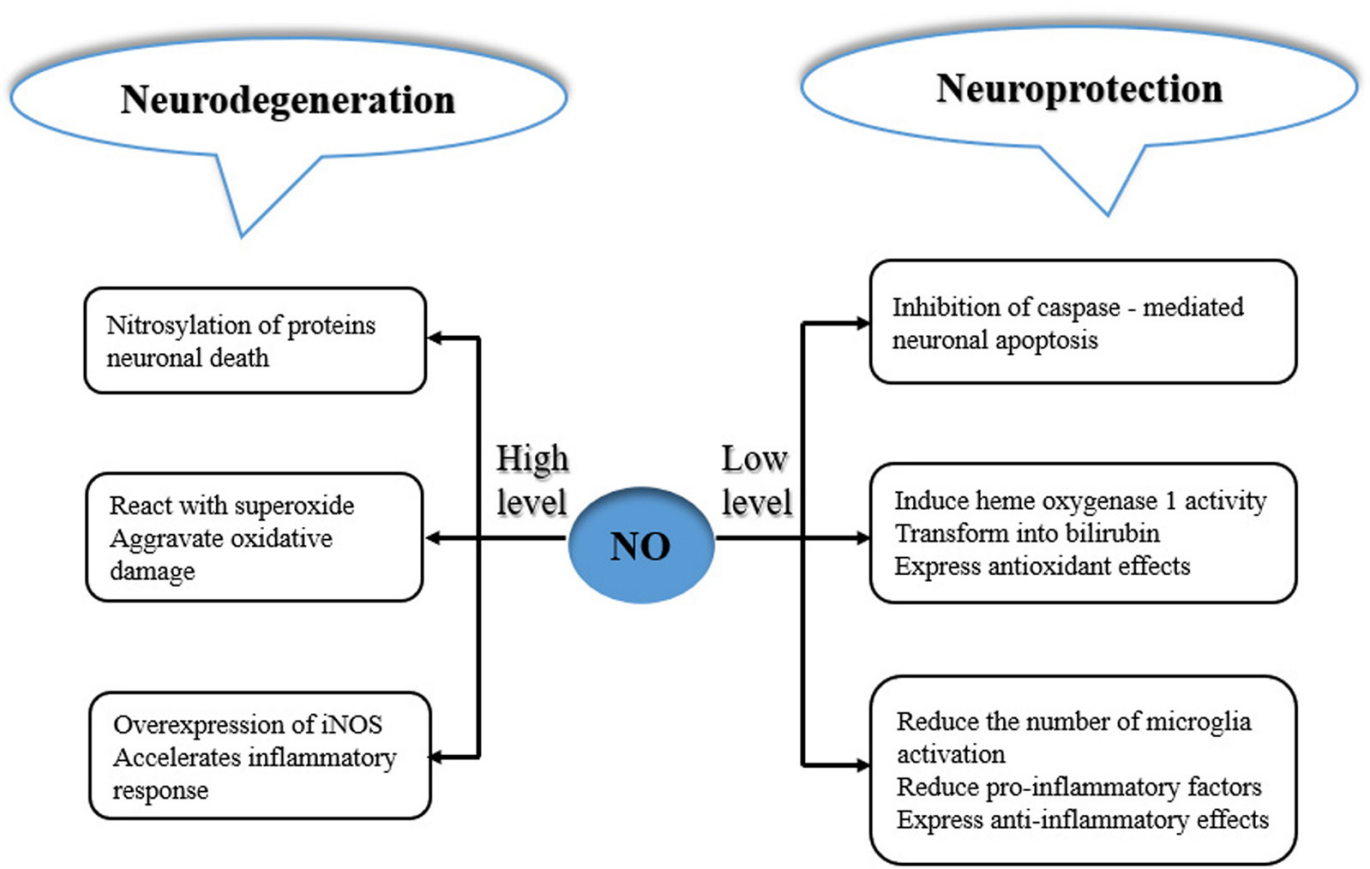
Neuroprotective and neurodegenerative aspects of NO including the pathways of their roles in development PD [29].

NO can inhibit iNOS expression by mediating the inactivation of the transcription factor, nuclear factor kappa B [54], thereby delaying the progression of inflammation or OS to protect the nerve cells. NO activates the cyclic adenosine monophosphate response element binding protein (CREB) and protein kinase (Akt) through the soluble guanylate cyclase (SGC)–cyclic guanylate (CGMP)–protein kinase G pathway. These proteins mainly have neuroprotective roles [55]. Nitric oxide can also induce the activity of heme oxygenase 1 (HO-1). HO-1 is responsible for the synthesis of biliverdin, which can be further converted into bilirubin with powerful antioxidant and anti-nitrous acid molecules [56]. The low concentration (physiological concentration) of NO released by the synthesis of nNOS can regulate the extracellular DA levels in the striatum and participate in the dopaminergic homeostasis of the striatum [55]. Thus, NO can protect neurons in various ways, and the concentration of NO is the main factor controlling the induction or inhibition of iNOS expression. Therefore, further studies should focus on controlling the supply of NO to investigate the role of NO in the progression of PD because NO can play a delaying or protective role in PD (Figure 5).

Compared with H_2_S, the control of NO concentration is difficult. Therefore, its applicability is limited. The relevant studies are few and the references are older. In medical research, NO mainly exists in the form of NO donors and NO gas. Common NO donors include nitroglycerin and sodium nitroprusside [57]. In the PD mouse model established using MPTP, the NO donor derivative of flurbiprofen [2-fluoro-*α*-methyl(1,1′-biphenyl)-4-aceticacid-4-(nitrooxy base) butyl ester] HCT1026 [55], it was found that compared with NO-free flurbiprofen, HCT1026 exhibited additional anti-inflammatory properties, which may be due to the inhibition of pro-inflammatory proteins (such as COX-2 and iNOS) and the inhibition of the release of pro-inflammatory cytokines (TNF-α) in activated macrophages. However, the NO donor still requires to solve the problem of NO release. Controlling the release amount and location can lead to an accurate NO effect. Along with NO donors, the research on NO gas has garnered much attention. The methods used include inhalation and microbubble transmission. Presently, inhaled NO is mainly used in the treatment of neonatal hypertension [58], and strict control is required during this treatment process. Owing to the toxicity and volatility of NO gas, the input amount of NO is greatly limited by direct inhalation of NO. Nitric oxide microbubbles [59] are also studied extensively in NO research. They have therapeutic effects in animal models of many diseases, including myocardial infarction, vascular plaque, and deep vein thrombosis [60]. However, no study has investigated its application in PD models. This is also a relatively new development in PD research.

## 5 Application of CO in PD

### 5.1 The synthesis, metabolism, and biological functions of CO

CO is a toxic, colorless, and odorless gas. Endogenous CO is mainly produced by heme oxygenase (HO) [61], which catalyzes heme. Three forms of heme oxygenase are present, among which HO-1 is an inducible enzyme, which can be induced by a hypoxic environment, endotoxin, and NO to increase its activity and play a role [62]. Similar to NO, CO activates SGC to participate in the signal transduction process; thus, different levels of CO and NO can regulate each other to exert physiological and pathological effects [63], thereby maintaining the homeostasis of the cellular environment of the body. Heme oxygenase 2 (HO-2) is a constitutively expressed enzyme, which is widely distributed, especially in the brain [64]. Heme oxygenase 3 (HO-3) is synthesized by the HO-1 gene, and its activity is low. Their relevant biological functions have not been confirmed yet. HO-1 and HO-2 are involved in the production of CO. Most of the CO produced in the body is excreted via respiration. CO has cytoprotective and therapeutic effects [65], including anti-inflammatory, anti-oxidative, anti-cancer, and anti-microbial properties, and it has protective effects against the prognosis of ischemia–reperfusion injury and organ transplantation [66].

### 5.2 Application pathways and related effects of CO in PD

CO acts as a regulatory molecule that affects various intracellular transduction pathways [61]. The generation of endogenous CO is mainly dependent on HO-1 and HO-2. HO-1 is an inducible catalytic enzyme, which is easily mobilized by the environment. Under physiological conditions, HO-1 is not produced in the central nervous system. HO can catalyze heme to generate CO, free ferrous iron (Fe^2+^), and biliverdin [56]. Under the action of biliverdin reductase (BVR), biliverdin is converted into bilirubin, and bilirubin can scavenge hydroxyl free radicals, singlet oxygen, and superoxide anion. Bilirubin, HO, and BVR have antioxidant and anti-inflammatory properties. Related studies have found that [65] the secretion of HO-1 can be monitored in patients with neurodegenerative diseases, including PD and Alzheimer’s disease. The HO-1/CO anabolic process plays a crucial role in OS and is a target for PD treatment. In 6-OHDA-induced C6 glioma cells [67], CO released by CORM-2 [68] attenuates cellular stress by decreasing apoptotic death signaling and increasing adaptive survival responses mediated by Nrf2, HO-1, and SODS death, which improves cell survival and increases the defense against OS in PD [69].

## 6 Application of H_2_ in PD

### 6.1 The characteristics and biological functions of H_2_

H_2_ is a mild inert gas, which is colorless, odorless, and non-toxic. It can rapidly diffuse into cells and tissues. It does not affect ROS [70] and plays a role in cell signal transduction; therefore, ingesting a certain amount of H_2_ has minimal adverse effects. H_2_ is spontaneously produced by gut bacteria during anaerobic metabolism in mammals. It is broken down by hydrogenase to provide electrons to produce energy. H_2_ can be ingested or consumed *in vitro* [71] by various methods, including hydrogen inhalation, drinking H_2_-dissolved water (hydrogen water), injecting H_2_-dissolved saline (hydrogen saline), using H_2_-rich saline for eyes, and increasing bacterial production of H_2_ in the gut. The therapeutic application of H_2_ was first proposed in 1975. However, owing to the lack of clinical application during that time and the lack of recognition of H_2_, it was considered a non-functional gas. In 2007, Shigeo Ohta et al. [72]. reported that H_2_ has selective antioxidant properties. It specifically neutralizes hydroxyl radicals (−OH) and peroxynitrite (ONOO^−^), and it does not decrease other ROS and NO, thus protecting the brain. The therapeutic application of H_2_ has garnered much attention since then [73]. H_2_ can directly decrease OS by reacting with strong oxidants and also indirectly decrease OS via the regulation of the expression of various genes [74]. H_2_ can regulate anti-inflammatory and mitochondrial energy metabolism and endoplasmic reticulum stress. It is involved in the immune system and the reduction of cell death [75]. It plays crucial roles in multiple systems and multiple organs and has therapeutic potential for many systemic diseases [71].

### 6.2 The role of H_2_ in PD

OS majorly contributes to the occurrence and progression of PD. H_2_ can rapidly diffuse into tissues and cells without affecting metabolic redox reactions and signal reaction substances [73]. H_2_ has good antioxidant activity and can react with strong oxidants in cells (including hydroxyl and nitrosyl free radicals) and decrease OS by regulating gene expression, thereby playing an anti-inflammatory and antiapoptotic role [73]. Previous studies have indicated that the intestinal flora of patients with PD has imbalanced and less exhaled H_2_ [76]. In MPTP-induced PD model mice, H_2_ water was ingested through the enteral route. H_2_ can attenuate the toxic effect of neurotoxins on dopaminergic neurons in the substantia nigra-striatum pathway and effectively decline movement disorders [76]. H_2_ is a gas with specific anti-OS effects in the PD rat model established by 6-OHDA [72]. It is recommended to drink water containing 50% saturated molecular H_2_ before and after undergoing the procedure. H_2_ can prevent the development of substantia nigra degeneration and also prevent its progress. After staining treatment, it was found that H_2_ can prevent the loss of dopaminergic cells, which indicated that H_2_ can delay the development and progression of PD [72].

H_2_ can be ingested using various methods, such as inhaling H_2_, drinking H_2_-dissolved water (H_2_-water), injecting H_2_-dissolved saline (H_2_-saline), taking an H_2_ bath, or dripping H_2_-saline into the eyes [77]. H_2_ is relatively safe for experimental or clinical application owing to its non-toxic nature. H_2_ is distinct from the abovementioned gas signaling molecules [78]. Owing to its curative effect and no adverse reactions, H_2_ has potential in the clinical treatment of PD.

## 7 Conclusion

To summarize, gas signaling molecules can exert biological functions in many systems of the body. They exhibit significant therapeutic effects in many diseases. However, owing to the biological characteristics of gas signaling molecules, including toxicity and easy diffusion, their applicability is greatly limited. Gases play a crucial role in PD through different mechanisms or signal transduction pathways. Although the mechanism underlying the role of some gases is still unclear, this article provides a basis for further research. Based on the characteristics of the abovementioned gases, exploring more effective application methods is crucial to improve the progress of PD treatment in the future.
